# Emergency surgical decompression for spontaneous spinal epidural hematoma in octogenarians: risk factors, clinical outcomes, and complications

**DOI:** 10.1007/s00701-022-05457-7

**Published:** 2022-12-26

**Authors:** Pavlina Lenga, Marilena Knittelfelder, Gelo Gülec, Karl Kiening, Andreas W. Unterberg, Basem Ishak

**Affiliations:** Department of Neurosurgery, Heidelberg University Hospital, University of Heidelberg, Im Neuenheimer Feld 400, 69120 Heidelberg, Germany

**Keywords:** Decompressive laminectomy, Neurological decline, Spontaneous spinal epidural hematoma, Octogenarians

## Abstract

**Purpose:**

Spontaneous spinal epidural hematoma (SSEH) is a rare but disabling disease. Although several cases have been reported in the literature, their treatment remains unclear, especially in patients with advanced age. We, therefore, aimed to describe the clinical outcomes of cervical SSEH in octogenarians with an acute onset of neurological illness undergoing laminectomy.

**Methods:**

Electronic medical records from a single institution between September 2005 and December 2020 were retrieved. Data on patient demographics, neurological conditions, functional status, surgical characteristics, complications, hospital course, and 90-day mortality were also collected.

**Results:**

Twenty-two patients aged ≥ 80 years with SSEH undergoing laminectomy were enrolled in this study. The mean Charlson comorbidity index was 9.1 ± 2.0, indicating a poor baseline reserve. Ten individuals (45.5%) were taking anticoagulant agents with a pathologic partial thromboplastin time (PTT) of 46.5 ± 3.4 s. Progressive neurological decline, as defined by the motor score (MS), was observed on admission (63.8 ± 14.0). The in-hospital and 90-day mortality were 4.5% and 9.1%, respectively. Notably, the MS (93.6 ± 8.3) improved significantly after surgery (*p* < 0.05). Revision surgery was necessary in 5 cases due to recurrent hematoma. Anticoagulant agents and pathological PTT are significant risk factors for its occurrence. Motor weakness and comorbidities were unique risk factors for loss of ambulation.

**Conclusions:**

Laminectomy and evacuation of the hematoma in octogenarians with progressive neurological decline induce clinical benefits. Emergent surgery seems to be the “state of the art” treatment for SSEH. However, potential complications associated with adverse prognostic factors, such as the use of anticoagulants, should be considered.

## Introduction

Spontaneous spinal epidural hematoma (SSEH) is a rare but disabling disease initially described in 1869 [17]. Its estimated incidence is approximately 0.1 patients per 100,000 [14] and accounts for less than 1% of spinal occupied lesions [12]. Due to the rarity of SSEH, the etiopathology and underlying mechanisms that cause severe neurological deficits remain unclear. The classical clinical picture encapsulates a surge of back pain, followed by symptoms caused by nerve root or spinal cord compression, resulting in neurological deterioration and neurological emergency [12, 15, 22]. The time frame between signs and symptoms that reflect neurological decline due to neural compression can last minutes to days; thus, a prompt diagnosis might be difficult and often delayed. Therefore, based on the clinical suspicion of SSEH, swift spinal magnetic resonance imaging (MRI) should be performed to verify the diagnosis, enabling prompt initiation of therapy.

Although some studies advocate that conservative management of SSEH in the presence of mild neurological symptoms can lead to good clinical outcomes [6, 12, 13], the gold standard, especially in the face of rapid deterioration of spinal cord dysfunction, is emergent surgical decompression and evacuation of the hematoma [20, 21, 23, 32]. The disease presents with a higher prevalence in the fourth and fifth decades of life, and in a world with an aging population growing faster than the total population, octogenarians can also suffer from this devastating condition. Predisposing factors including hypertension [2], coagulopathy, anticoagulation [11], and vascular malformations [34] have been suggested, which are conditions that mainly manifest in the aging population. The literature relies unreassuringly solely on case reports; a systematic analysis reporting on beneficial therapies and clinical outcomes is still missing. For instance, in a recent review on SSEH, the authors advocated that spinal canal decompression via laminectomy irrespective of the affected region might be the mainstay of treatment resulting in significant improvements of the preoperative motor deficits as defined by the ASIA [26, 33]. Notwithstanding, geriatric population and especially octogenarians are playing mostly a marginal role so far in clinical studies. Considering their poor baseline history, elderly patients might be at a higher risk of poor outcomes from SSEH, which mainly includes cardiovascular diseases. However, despite some case reports [9, 28], there is no robust evidence on the optimal treatment for this subset of patients considering their unique needs due to the multitude of age-related variables that might impact outcomes.

Owing to the lack of clinical evidence on this topic, this study sought to provide a guide for more efficient management of cervical SSEH in patients aged ≥ 80 years with an acute onset of neurological decline by assessing the clinical course and morbidity and mortality rates, and determining potential risk factors for patients losing their ability to walk after surgical decompression during a long-term follow-up.

## Methods

### Study design and inclusion criteria

**Fig. 1:**
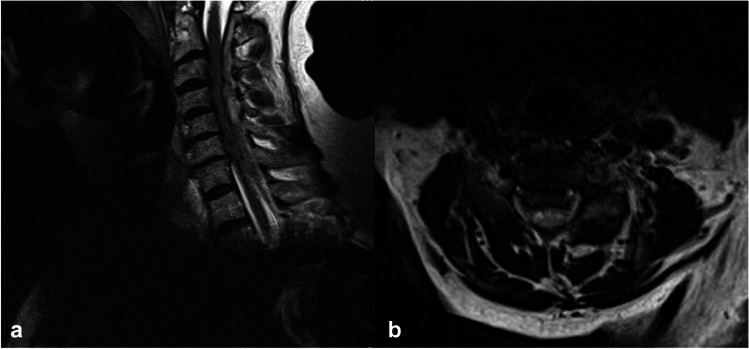
An 81-year-old man suffered from a sudden-onset of posterior neck pain and then, he developed paraplegia and anesthesia below T4. On sagittal T2-weighted images (**a**), the hematoma was located on the portion dorsal to the dural sac on the cervical spine (C3–C6). On the axial T2-weighted image (**b**), the hematoma was located on dorsal portion around to dural sac, and the dural sac was severely compressed

Clinical and imaging data were retrospectively collected over a 16-year period (September 2005–December 2021) from our institutional database. This study was approved by the local ethics committee of our institution (approval number 880/2021) and conducted in accordance with the Declaration of Helsinki. The requirement for informed consent was waived because of the retrospective nature of the study. Patients aged ≥ 80 years with SSEH across the cervical spine who presented to our institution with acute neurological decline were consecutively enrolled. The diagnosis was based on MRI (Fig. 1). Due to the presence of acute neurological deficits, all patients underwent surgical decompression via laminectomy and hematoma evacuation within the first 24 h of the manifestation of the first symptom (Fig. 1). None of the patients received conservative management. Patients under anticoagulants received antidotes before surgery based on the German Guidelines to interrupt the anticoagulation effects [29]. The doses of the antidote medication were decided by an experienced anesthesiologist according to the current guidelines, which are based on the baseline characteristics (such as age, BMI, and renal function) of each patient. Since patients presented to our department with neurological decline or motor deficits, we initially performed a CT scan to identify if the cause of the decreased neurological condition was attributable to cranial pathology, such as a hematoma. In all of the examined cases, intracranial pathology was absent. To further clarify the cause of the patients’ clinical condition, an MRI of the total spinal cord was performed. A meticulous study of the images together with neuroradiologists and spine surgeons was conducted and the pathology in the cervical spine of these cases was identified, while no further hemorrhages were seen. The exclusion criteria were as follows: (1) age < 80 years; (2) hematoma related to trauma or spinal surgery; (3) hematoma associated with spinal punctures/catheters, arteriovenous malformation, or tumor; (4) short-term follow-up (less than 1 month); (5) previous spinal surgery in the past 3 months; and (6) concomitant intracranial pathologies or further spinal hemorrhage—since these would have a significant impact on the patients’ outcome.

### Patient characteristics

Patient demographics, comorbidities, American Society of Anesthesiologists (ASA) scores, duration of surgery, number of treated spinal levels, perioperative and postoperative complications, hospital length of stay (LOS), intensive care unit (ICU) stay, readmission, reoperation, and mortality were retrieved from patients’ electronic medical records. Partial thromboplastin time (PTT; normal range 25–35 s) was also extracted from patient laboratory records. Comorbidities present before surgery were assessed using the age-adjusted Charlson comorbidity index (CCI) [8, 7]. The CCI was calculated for each patient and classified as no comorbidity (CCI = 0), minimal comorbidity (CCI = 1 or 2), moderate comorbidity (CCI = 3–5), or severe comorbidity (CCI > 5). The pre-treatment neurological condition was assessed using the motor score (MS) of the American Spinal Injury Association (ASIA) impairment grading system (MS = 0, no muscle strength; MS = 100, healthy). All patients presented with an acute onset of neurological decline; hence, posterior decompression via laminectomy was performed in the first 24 h. Decision-making was guided by presenting neurological status (MS), concomitant underlying pathologies, extent of the pathology, prognosis of the disease, and the discretion of an experienced treatment team of neurosurgeons, neuroradiologists, and anesthesiologists. Two experienced spine surgeons (BI and KK) performed the final decision-making mainly based on the patients’ clinical condition, with a special focus on the neurological decline, as well as on the mass effect of the hematoma as shown in the MRI. Both surgeons jointly decided on surgical decompression via laminectomy and evacuation of the hematoma, aiming to preserve or improve the patients’ neurological condition post-surgery. Routine clinical follow-up examinations were performed 3 months after surgery.

### Surgical technique

Surgeries were performed according to our institutional standards. All surgeries were performed by two experienced surgeons (KK and BI) under general anesthesia. A three-pin head holder was affixed to the patient’s head, and the patient was in prone position on the operating room table, with the trunk supported by padded chest rolls or a four-poster frame. First, a midline incision was made to expose bony structures. Monopolar electrocautery was used to continue the dissection in the midline. The ligamentum nuchae was divided, and the dissection continued to split the splenius capitis fascia. The plane between the semispinalis capitis musculature was then entered. The spinous processes were resected, and a partial laminectomy was performed with a high-speed drill. The remaining lamina was then thinned using a high-speed drill, and the laminectomy was completed with angled punches. The blood, which for the most part was coagulated, was removed, and no particular difficulty was encountered in obtaining hemostasis. The dura mater appeared normal, and no active hemorrhage was observed within the subdural space. No vascular malformations or tumors were detected during surgery.

The surgical approach in case of recurrent hematomas was performed by the same surgeons of our study group (BI and KK). The indication of reoperation was based once again on the patients’ neurological condition and on the MRI of the cervical cord. If the patients suffered from new motor deficits after surgery and re-hematoma causing substantial mass effect on the dural sack was seen in MRI, re-surgery was performed with the aim to identify the cause of re-bleeding. In all cases with postoperative SEH, the previous incision and access were re-explored.

### Statistical analysis

Categorical variables were presented as numbers and percentages. Continuous variables are presented as mean ± standard deviation, and the Shapiro–Wilk test was used to verify whether their distribution was normal. Baseline characteristics, duration of surgery, number of treated spinal levels, perioperative and postoperative complications, LOS, ICU stay, readmissions, reoperations, and mortality were compared groupwise using independent *t*-tests for continuous variables and chi-squared tests for categorical variables. The Wilcoxon rank test was used to evaluate changes in neurological status (MS) at discharge. In the second-stage analysis, binary logistic regression was performed to identify the risk factors for hematoma recurrence after surgery and the loss of ambulation. Statistical significance was set at *p* ≤ 0.05.

## Results

### Epidemiological data and baseline characteristics

Over a period of 15 years, 22 patients aged ≥ 80 years with SSEH undergoing surgical decompression via laminectomy were enrolled in this study. The mean age was 84.3 ± 3.1 years with female predominance (13/22, 59.1%). The mean CCI was 9.1 ± 2.0, indicating a poor baseline reserve. Cardiovascular diseases such as arterial hypertension, myocardial infarction, coronary heart disease, and atrial fibrillation were the most prevalent (*n* = 18, 50%; *n* = 11, 50%; *n* = 11, 50%; and *n* = 10, 45.5%, respectively). Ten individuals (45.5%) were taking anticoagulant agents and had a pathologic PTT of 46.5 ± 3.4 s. Notable decline was seen in neurological condition, as defined by a mean MS of 63.8 ± 14.0. All SSEHs were located in the dorsal space of the cervical spine. The patient characteristics are shown in Table 1.

**Table 1:** Baseline characteristics

Characteristic	Value
Number of patients	22
Age, years (mean, SD)	84.3 (3.1)
Sex (*n*, %)	
Male	9 (40.9)
Female	13 (59.1)
Body mass index, kg/m^2^ (mean, SD)	24.4 (2.0)
Comorbidities	
Age-adjusted CCI score (mean, SD)	9.1 (1.4)
Arterial hypertension (*n*, %)	18 (81.8)
Myocardial infarction (*n*, %)	11 (50.0)
Coronary heart disease (*n*, %)	11 (50.0)
Atrial fibrillation (*n*, %)	10 (45.5)
Heart failure (*n*, %)	7 (31.8)
COPD (*n*, %)	3 (13.6)
Diabetes mellitus type II (*n*, %)	4 (18.2)
Renal failure (*n*, %)	9 (40.9)
Liver disease (*n*, %)	3 (13.6)
Gastrointestinal ulcer (*n*, %)	5 (22.7)
TIA/stroke (*n*, %)	4 (18.2)
Malignancy (*n*, %)	11 (50.0)
Dementia (*n*, %)	3 (13.6)
Previous spinal surgery (*n*, %)	3 (13.6)
ASA class (*n*, %)	
II	1 (4.5)
III	17 (77.3)
IV	4 (18.2)
V	
Preoperative MS (mean, SD)	63.8 (14.0)
Anticoagulants (*n*, %)	10 (45.5)
PTT, s (mean, SD) *	46.5 (3.4)

*ASA*, American Society of Anesthesiologists; *CCI*, Charlson comorbidity index; *COPD*, chronic obstructive pulmonary disease; *MS*, motor score of the American Spinal Injury Association grading system; *SD*, standard deviation; *TIA*, transient ischemic attack; *PTT*, partial thromboplastin time

^*^Patients receiving anticoagulation therapy

### Surgical characteristics and clinical course

As shown in Table 2, the mean duration of surgery was 151.8 ± 38.1 min with a mean blood loss of 517.5 ± 433.2 mL. The mean number of decompression levels was 2.4 ± 1.1. Intraoperative blood transfusion was necessary in 4 cases (1.2%). During hospitalization, one patient died due to acute heart failure, while 2 more patients (9.1%) died within 90 days due to the same cause. The neurological status improved significantly after surgery, with a postoperative mean MS of 78.0 ± 13.3 (Table 3). The mean follow-up period was 26.8 ± 4.5 months, and no additional fusion surgery due to secondary instability was necessary.

**Table 2:** Perioperative and postoperative surgical characteristics and clinical course of 22 patients who underwent decompression surgery

Characteristic	Value
Surgical duration, min	151.8 (38.1)
Number of levels decompressed	2.4 (1.1)
Estimated blood loss, mL	517.5 (433.2)
Blood transfusion (*n*, %)	4 (18.2)
Hospital stay, days	9.0 (4.5)
ICU stay, days	2.1 (1.4)
Mortality	
In-hospital (*n*, %)	1 (4.5)
90-day (*n*, %)	2 (9.1)
30-day readmission (*n*, %)	1 (4.5)
Postoperative MS	78.0 (13.3)

Values represent the mean (SD), except where otherwise indicated

*ICU*, intensive care unit; *MS*, motor score of the American Spinal Injury Association grading system

**Table 3:** Occurrence of adverse events in octogenarian patients (*N* = 22) who underwent decompression surgery

Event	Number of patients (%)
Deep wound infection	2 (9.1)
Acute heart failure	3 (13.6)
Septic shock	1 (4.5)
Pneumonia	3 (13.6)
Ileus	1 (4.5)
Urinary tract infection	2 (9.1)
Bleeding	2 (13.6)

### Complications, revision surgery, and risk factors for the loss of ambulation

The most prevalent complications were recurrent hematomas (27.2%) and pneumonia (13.6%). A detailed breakdown of the complications is presented in Table 4. Motor weakness and the presence of comorbidities were unique risk factors for loss of ambulation, whereas arterial hypertension, anticoagulants, pathologic PTT, surgical duration, extension of surgery, hospital or ICU stay, and complications were not (Table 5). Revision surgery was required in five cases of recurrent hematoma due to secondary neurological decline. In all five cases, small vessels were identified as the cause of bleeding and were coagulated without any difficulty. One drain tube was placed over the laminectomy site as close as possible to the dura mater. Negative vacuum pressure was applied to the drain within 10 min after the commencement of wound closure in all 5 cases. Drains were removed without the occurrence of any complications 2 days after surgery. Patients regained their well-being and were able to walk after surgery. Logistic regression analysis was conducted to identify the potential risk factors for the occurrence of postoperative spinal hematoma. Of note, an elevated PTT and preoperative use of anticoagulants were significant risk factors for the occurrence of postoperative hematoma (Table 6).

**Table 4:** Comparison of baseline (before surgery), discharge neurological condition, and functional status scores

	Decompression Baseline *N* = 22	Decompression Discharge *N* = 22	*p* value
MS	63.8 (14.0)	78.0 (13.3)	** < 0.001**

All data are presented as mean (SD)

*MS*, motor score of the American Spinal Injury Association grading system

*p*-values significant should be denoted as bold

**Table 5:** Risk factors associated with the inability to walk after surgery

Risk factor	OR (% 95 CI)	*p* value
Age-adjusted CCI score	1.7 (1.1–4.9)	**0.032**
Preoperative MS	1.3 (1.1–1.8)	**0.004**
PTT	1.1 (0.9–1.3)	0.876
Duration of surgery	1.1 (1.0–1.3)	0.986
Estimated blood loss	1.0 (0.9–1.1)	0.984
Number of levels decompressed	0.3 (0.1–0.9)	0.089
Length of ICU stay	0.3 (0.2–1.1)	0.201
Length of hospital stay	1.1 (0.8–1.6)	0.893
Complications	1.5 (1.0–2.7)	0.065

*CCI*, Charlson comorbidity index; *CI*, confidence interval; *ICU*, intensive care unit; *MS*, motor score of the American Spinal Injury Association grading system; *OR*, odds ratio; *PTT*, partial thromboplastin time

*p*-values significant should be denoted as bold

**Table 6:** Factors associated with re-bleeding after surgery

Risk factor	OR (% 95 CI)	*p* value
PTT	1.2 (1.0–2.2)	**0.017**
Anticoagulants	1.7 (1.1–3.2)	**0.012**
Duration of surgery	1.8 (1.0–2.2)	0.171
Estimated blood loss	0.8 (0.3–1.4)	0.423
Number of levels decompressed	1.2 (0.9–1.8)	0.747

*PTT*, partial thromboplastin time

*p*-values significant should be denoted as bold

## Discussion

To the best of our knowledge, this is the first study with such a large cohort of octogenarians to examine the clinical course of surgical management for cervical SSEH. We assessed morbidity and mortality rates and determined potential risk factors for both loss of ambulation and occurrence of postoperative SSEH exclusively in octogenarians undergoing spinal decompression less than 24 h after the first signs of neurological deterioration. Octogenarians constitute a frail cohort with an age-adjusted CCI of 9.1, and almost 50% were receiving anticoagulant agents with an increased PTT of 46.5 s. The in-hospital mortality rate was 4.5%, while the 90-day mortality increased by 9.1%. During the follow-up period of more than 2 years (26.8 months), no deaths occurred. Interestingly, significant improvements in neurological deficits, as measured by MS, were seen at discharge, and 63.6% regained their ability to walk after surgery. The remaining patients recovered substantially and regained independence in their daily activities after rehabilitation. Notably, higher rates of comorbidities and higher grades of preoperative motor weakness were significant risk factors for loss of ambulation after surgery. Revision surgery due to postoperative SSEH required five patients to use anticoagulants in-house. Logistic regression analysis revealed that the use of anticoagulant agents and increased PTT were significantly associated with postoperative occurrence of SSEH.

### Review of literature

Miscellaneous factors such as arterial hypertension, anticoagulant agents, lifting, and vascular anomalies have been hypothesized to predispose patients to SSEH [2, 11, 34]. In the present study, despite the generally high rates of comorbidities, over 80% of patients presented with arterial hypertension and almost 50% were under anticoagulation due to a cardiovascular disease. Liao et al. (2009) conducted a retrospective study of 35 patients with a mean age of 42.6 years and reported that arterial hypertension was present in 34.3% of the cases, while only one patient received an anticoagulant [20]. No significant association was found between hypertension or anticoagulant use and clinical outcomes. In contrast, Nitta et al. (2021) in their retrospective analysis of 30 patients with a mean age of 67 years (range 62–78 years) showed that both higher rates of underlying diseases, as determined by ASA, and preoperative consumption of anticoagulant agents were significant risk factors for worse outcomes [25]. Groen and Ponssen (1990), in a review and meta-analysis of 199 cases, also expressed the notion that there might be a potential relationship between arterial hypertension, anticoagulant use, and the coexistence of SSEH [12], a phenomenon most frequently observed in older patients because of their cardiovascular profile. In accordance with these findings, Shin et al. (2006) postulated that both arterial hypertension and anticoagulation might contribute to higher rates of bleeding, although a causative relationship could not be established [27]. Some case reports also indicated that patients receiving oral anticoagulants, such as dabigatran or rivaroxaban, have a higher risk of the occurrence of SSEH ([1, 16]. It should be noted that the aforementioned studies mainly examined younger patients, while patients aged ≥ 80 years were either marginalized or ignored. According to the findings of the present study dedicated to octogenarians, both arterial hypertension and anticoagulation might be two key factors contributing to the occurrence of SSEH; however, these results should be considered with caution owing to the small sample size. Undoubtedly, increased awareness is warranted among emergency physicians confronted with such cases. Unreassuringly, neither parameter significantly affected the clinical outcomes.

It is well known that octogenarians are impaired due to higher rates of comorbidities, which is why a surgical procedure for spinal pathology is currently viewed with reluctance due to potential postoperative complications that may also lead to death. However, in the presence of acute onset of neurological deficits, surgery is inevitable in such a debilitating cohort. According to the findings of the present study, each patient underwent surgical decompression of the cervical spinal canal via laminectomy and hematoma evacuation of at least two segments less than 24 h after the initial presentation of neurological decline. Most importantly, a significant improvement in motor deficits was observed at discharge. Unique risk factors for loss of ambulation were higher rates of comorbidities and higher degrees of neurological deficits on admission. After surgery, eight patients were unable to walk with an initially complete paraplegia, whereas a slight improvement was observed. Therefore, the patients were transferred to a rehabilitation clinic. At the latest follow-up, the patient had recovered fully. Consistent with these findings, Liao et al. (2009) highlighted the importance of swift surgical management after neurological deterioration. In particular, they showed that patients undergoing surgery within 48 h after the first neurological signs experienced significantly better neurological recovery compared to those treated after 48 h [20]. Factors such as age, sex, location, and extent of surgery did not significantly affect the outcomes [20]. Similarly, Zhong et al. (2011) concluded that early surgery after symptom onset might be a key factor for better recovery. Additionally, they stated that worse preoperative neurological status was correlated with less favorable outcomes, whereas these patients fully recovered at the latest follow-up, as also shown in the present study [32]. In another study of 10 older patients with SSEH (range 72–84 years), emergent decompression and evacuation of the hematoma led to substantially good recovery rates [10]. In line with the abovementioned studies, Yan et al. (2022) also found that preoperative severe neurological deficits and extended paraplegia time of more than 12 h were significantly correlated with a worse prognosis [30]. Therefore, although a general consensus concerning the optimal management of such cases is still debatable, we feel that emergent decompression gray might be a critical pillar for the therapy of SSEH in such a frail cohort.

The mechanisms underlying SSEH are not yet fully understood. To date, two mechanisms have been proposed for this phenomenon. Beatty and Winston (1984) advocated that the vertebral venous plexus (a valveless low-pressure system) is associated with the abdominal and venous systems; thus, an increase in intraabdominal or intrathoracic pressure can lead to an elevated intraspinal venous plexus [3]. This may result in a rupture of the vessels of the epidural venous plexus, mainly those in the dorsal space where most SSEHs are found [3]. However, considering that intrathecal pressure is higher than epidural venous pressure, an epidural arterial source is thought to be the inciting event, especially in the presence of arterial hypertension or coagulopathy [12, 21]. In the present study, almost all individuals presented with arterial hypertension and half of them were under anticoagulant agents; therefore, these conditions might contribute to the rupture of an arterial epidural vessel, thus causing the hematoma. Nevertheless, all SSEHs were in the dorsal epidural space. Therefore, the venous vessel theory might be the impetus for SSEH. We feel that both mechanisms should be further pursued in future studies to shed light on this still poorly understood topic.

In a retrospective study of 30 patients with SSEH, Zhong et al. (2011) reported deaths (16.7%), of whom 4 had a cervicothoracic SSEH (2 underwent surgery and 2 underwent conservative management) and died due to respiratory failure and 1 due to gastrointestinal infection immediately after surgery [32]. In contrast, Liao et al. (2009) found a lower mortality rate of 5.7%, which was not surgery-related [20]. This discrepancy between the studies might be attributable to the fact that, in the series from Zhong et al. (2011), patients were treated conservatively and death was inevitable due to the rapid disease progression. As shown in previous studies, disease-related mortality ranges from 6 to 8% and is highly correlated with cervical or cervicothoracic hematomas [12, 30], which is in line with the findings of the present study exclusively in octogenarians with cervical SSEH. In our series, one patient died due to acute heart failure, whereas two died within 90 days due to severe pneumonia. None of the patients died during the surgical procedure.

Although the use of anticoagulant agents significantly affected the clinical outcome, we showed that a coagulation disorder as measured by PTT due to the use of anticoagulant agents was significantly associated with re-bleeding immediately after surgery. There are still contradictory results concerning the causal relationship between anticoagulant use and re-bleeding after spinal surgery. In their retrospective study of 3729 patients undergoing spinal surgery, Yi et al. (2006) concluded that postoperative hematoma was significantly associated with anticoagulation therapy [31]. Lawton et al. (1995), in a retrospective analysis of 30 patients with a mean age of 48 years, asserted that postoperative spinal hematoma is correlated with both previous spinal surgery and anticoagulation medication [19]. In agreement with these findings, Kou et al. (2002) found that coagulopathy and increasing age constitute to higher risk of postoperative spinal hematoma [18]. Herein, it should be emphasized that our patients received antidotes to eliminate the anticoagulation effects of the anticoagulant agents according to the German Guidelines [29]. Therefore, it raises the question why the use of such medication poses a risk factor for the recurrence of the SSEH. One potential explanation may be that the geriatric population and especially octogenarians, due to their pure baseline history with multiple prolonged comorbidities, may require discontinuation intervals since their renal function is substantially impaired (creatinine clearance of less than 30 mL/min) [24]. Previous studies have stated that impaired renal function is a significant risk factor and prolongs the effects of anticoagulant agents despite discontinuation before surgery or the use of antidotes to counter their effect [4, 5]. Noteworthily, according to the findings of the present study, over 40% of the examined patients presented with a chronic renal failure, while all the 10 patients who received anticoagulant agents suffered from impaired renal function. Thus, we feel that these findings support the hypothesis, as it was also previously shown that impaired renal function serves as an important confounding factor for a prolonged effect of the anticoagulants even after the administration of antidotes. Further research is warranted since octogenarians are mostly precluded from such studies, presumably due to their multiple underlying diseases related with worse outcomes [4]. Nevertheless, we believe that, especially in the case of octogenarians, a meticulous preoperative evaluation with a sufficient record of the underlying diseases should be conducted. Therefore, surgeons will be alerted in case of a secondary postoperative neurological deterioration, and an emergent surgery can be performed to prevent further worsening of the clinical outcome or even death, especially in cases with cervical hematoma.

### Limitations

The main strength of the current study is that it is the first to systematically examine the clinical course and outcomes exclusively in octogenarians with such a rare disease. However, this study has some limitations. First, a relatively small cohort of patients was investigated. However, since the previous data come mainly from case reports, we feel that our findings provide a real-world picture of the diseases. Second, selection bias might have been present because of the retrospective study design. Third, a multivariate analysis for loss of ambulation could not be performed because of the limited number of cases. Larger studies are warranted to elucidate the mechanisms and roles of anticoagulant agents in the pathogenesis of SSEH.

## Conclusions

Our findings showed that emergent surgical decompression of the cervical canal might be the key tool to treat this subset of patients with an acute onset of neurological deficits since it preserves functional outcomes. However, preoperative neurological deficits and rates of comorbidities should be considered since they are strongly associated with disease prognosis and loss of the regaining of their ambulation. Since anticoagulant agents are significant predictors of recurrence of the hematoma, there is an imperative to try to eliminate the effect of such medication even though that is hampered by patients impaired renal function. Potential complications correlated with the risk baseline profile of the patients should be clearly discussed with the patient and their relatives.

## Data Availability

The datasets generated and/or analyzed during the current study are available from the corresponding author on reasonable request.
